# Theta-Gamma Coding Meets Communication-through-Coherence: Neuronal Oscillatory Multiplexing Theories Reconciled

**DOI:** 10.1371/journal.pcbi.1005162

**Published:** 2016-10-14

**Authors:** Douglas McLelland, Rufin VanRullen

**Affiliations:** CerCo, Université de Toulouse Paul Sabatier, CNRS, France; École Normale Supérieure, College de France, CNRS, FRANCE

## Abstract

Several theories have been advanced to explain how cross-frequency coupling, the interaction of neuronal oscillations at different frequencies, could enable item multiplexing in neural systems. The communication-through-coherence theory proposes that phase-matching of gamma oscillations between areas enables selective processing of a single item at a time, and a later refinement of the theory includes a theta-frequency oscillation that provides a periodic reset of the system. Alternatively, the theta-gamma neural code theory proposes that a sequence of items is processed, one per gamma cycle, and that this sequence is repeated or updated across theta cycles. In short, both theories serve to segregate representations via the temporal domain, but differ on the number of objects concurrently represented. In this study, we set out to test whether each of these theories is actually physiologically plausible, by implementing them within a single model inspired by physiological data. Using a spiking network model of visual processing, we show that each of these theories is physiologically plausible and computationally useful. Both theories were implemented within a single network architecture, with two areas connected in a feedforward manner, and gamma oscillations generated by feedback inhibition within areas. Simply increasing the amplitude of global inhibition in the lower area, equivalent to an increase in the spatial scope of the gamma oscillation, yielded a switch from one mode to the other. Thus, these different processing modes may co-exist in the brain, enabling dynamic switching between exploratory and selective modes of attention.

## Author Summary

There is a growing consensus that neuronal oscillations constitute a fundamental computational mechanism in the brain. Beyond this, recent experimental evidence has highlighted interactions between oscillations at high and low frequencies (e.g. gamma oscillations, 40–80 Hz, are modulated by theta oscillations, 4–10 Hz), and two major theories have developed regarding the functional role of this kind of cross-frequency coupling. Here, we present a computational modelling study of these theories with strong implications for biological studies. Firstly, we demonstrate for the first time that each of these theories is physiologically plausible, in that they can be implemented in a spiking network model with parameters guided by experimental data. Secondly, we show that they are each computationally useful, able to overcome a feature-binding ambiguity in a presented stimulus. Finally, we implement both theories within a single network model, and find that only a single parameter change is required to switch between the two processing states. This leads to the exciting new proposal that both theories may be correct, both implemented in the brain, with dynamic switching between modes according to processing and attentional requirements.

## Introduction

Neural systems continually process multiple information sources at a time, such as the various items in a visual scene, or the components of an episodic memory. It is widely accepted that the activity of different groups of neurons (assemblies or ensembles) represents the distinct items, and a general problem faced by such systems is that of overlap in the neural representations. In a system such as the hippocampus, where representation is apparently distributed across the network, ensembles must literally overlap. In more spatially structured systems, such as the retinotopic early visual system, the overlap arises because cells at later levels of the processing hierarchy have large receptive fields, potentially encompassing multiple stimulus items. This overlap creates an ambiguity in the signal, a potential confusion of the stimulus features or active neurons attributed to each of the different objects. It has long been recognized that one solution to this ambiguity would be to separate the activity of the different neuronal ensembles in the temporal domain [1], and that neuronal oscillations present a mechanism whereby this might be achieved [2,3].

In recent years there has been growing interest in cross-frequency coupling and the computations made possible by the interaction of oscillations at different frequencies[4,5]. At present, two major theories have been proposed, each requiring the interaction of a fast (gamma, 30–80 Hz) and a slow (alpha, 8–14 Hz, or theta, 3–8 Hz) oscillation, and each developed on the basis of experimental evidence within one system, but purporting to potentially describe a more general, fundamental computational mechanism in the brain. In this study, our aim was to test whether each of these theories is physiologically plausible, by implementing them within a unique network model inspired by physiological data, and to gain additional insights into the theories by doing so.

**Fig 1:**
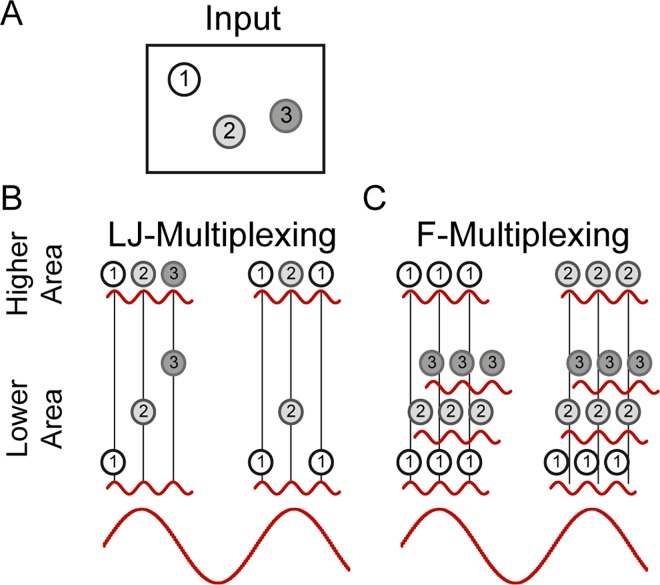
Comparative illustration of multiplexing theories. **A.** Consider a simple scene comprising 3 discrete objects of different saliency. **B.** LJ-multiplexing. Multiple Items are processed across consecutive fast cycles in decreasing order of saliency, and this sequence is repeated over slow oscillatory cycles. Note that items of low saliency may not be processed at all. **C.** F-multiplexing. Within any given slow cycle, only a single item is represented, repeated across multiple fast cycles. Across slow cycles, the represented item may switch, due to changes in the stimulus or top-down attentional mechanisms altering relative stimulus saliency.

The first of these theories (Fig 1B) was initially developed on the basis of observed activity patterns in the rodent hippocampus [6–8], and termed the ‘theta/gamma discrete phase code’. The theory has subsequently been extended to include a role in sensory systems [9–11] in which case experimental evidence suggests that the theta oscillation might be replaced by a slightly faster alpha oscillation. According to this theory, neurons participating in a given ensemble fire together within a single gamma cycle, with the inhibitory portion of the gamma cycle yielding a temporal separation before the activation of the next ensemble. The slow oscillation (theta in the hippocampus, alpha in the early visual system) structures the order of activation of the ensembles, because as the oscillatory inhibition fades on each cycle, more strongly activated ensembles are systematically activated earlier [12,13]. This ordering would reflect the natural ordering of items in a memory system, or the relative salience of items presented to a sensory system [9]. Depending on the ratio between fast and slow oscillatory frequencies as well as the “duty cycle” of the slow oscillation [9], between two and seven or more items can be multiplexed in this way. We will refer to this theory, for its principal developers Lisman and Jensen, as LJ-multiplexing.

The second theory (Fig 1C) was developed on the basis of experimental evidence from the visual system [14–17]. In this case, items are not separated on the discrete cycles of a common gamma oscillation. Instead, the activity of each ensemble is modulated by its own localized gamma oscillation, and it is the phase difference between these distinct oscillations that enables the selection and unambiguous processing of one individual item. Downstream neuronal regions selectively process one item at a time through the synchronization of their own local gamma oscillation to that of the item in question, referred to as ‘communication-through-coherence’ (CTC). That is, although item representations overlap temporally in the lower area, only a single representation is active at a time in the higher area. As such, the system described supports a mechanism of selection or attention. While the original CTC theory focussed on the role of gamma oscillations and the sustained selection of a single item [14], it was subsequently extended to consider slow (theta or alpha) oscillations as well [16,17]. In this case, the slow oscillation plays the important role of regularly breaking interareal synchrony, providing a system reset that enables, potentially, processing of different items over time. We will refer to this theory (to make clear the distinction from the original gamma-only CTC theory) as F-multiplexing, for its principal developer Fries.

Both of these theories have been presented as potentially generalized mechanisms of processing in the brain, although here for simplicity we shall focus on their potential role in perceptual processing. At first sight, the two theories appear incompatible, since one of them depends on the selection of a unique item (F-multiplexing) while the other maintains multiple items in near-simultaneity (LJ-multiplexing). Each theory has received support from experimental evidence in this context. In particular, for F-multiplexing, recent electrophysiological results (local field potential and single-unit recordings) have demonstrated modulation of interareal gamma coherence by attention, and theta-frequency fluctuations in gamma coherence [18–20], exactly as predicted by the theory. For LJ-multiplexing, evidence at the neuronal/network level relates mostly to the hippocampal system [21–24], although modulation of gamma activity by the phase of alpha activity has recently been demonstrated in the primary visual cortex [25] (this latter finding is equally supportive of F- as of LJ-multiplexing). On the other hand, certain behavioural-level results hint rather at LJ-multiplexing in visual processing. For example, the time required for a difficult visual search task increases with the number of distracter items. If interpreted as an indication of serial processing of individual items in turn (although this remains a controversial field *e*.*g*. [26]), these studies typically suggest processing times ranging from 20 to 60 ms per item [27], values consistent with LJ-multiplexing at one item per gamma cycle (around 25 ms), rather than F-multiplexing at one item per theta cycle (100–200 ms).

In order to test the mechanistic bases of these two multiplexing theories under physiological constraints, we developed a network model processing discrete stimulus objects. The network had no specific function other than to decompose and represent its visual input in as faithful a way as possible. In particular, we measured the temporal overlap between output neural responses coding for the different input stimuli, since this overlap could be construed as a failure to segregate input objects (e.g., yielding illusory conjunctions between features from different objects). Both LJ- and F-multiplexing modes have been proposed as candidate mechanisms to prevent such temporal overlap. We found that both LJ- and F-multiplexing modes can readily be implemented by a physiologically inspired network of spiking units, with a lower area establishing the temporal structure of the multiplexed signal, and a higher area decoding it. Secondly, the two operating modes are mechanistically much more closely related than might at first be apparent, and a single physiologically valid parameter shift (increasing the amplitude of global inhibition in the lower area, equivalent to the inclusion of a longer-range gamma oscillation) can drive a switch between operating modes. This raises the intriguing possibility that these two different theories are actually both correct and co-exist in the brain. In that case, they may serve to implement an exploratory mode (LJ-multiplexing) and a selective processing mode (F-multiplexing), and the system could dynamically switch between them as required.

## Results

In what follows, we first describe our model, and the nature of the fast and slow oscillations. We then describe a set of simulation results showing that, with changes in a single parameter, the network can enter either the F- or LJ-multiplexing states (a second parameter change is required to increase the number of items represented in LJ-multiplexing). We go on to characterise and describe in detail what is actually happening in the network in each case. We then present simulations exploring further details for each state (for F-multiplexing, the role of the slow oscillation and the potential to switch the selected item over slow cycles; for LJ-multiplexing, the potential to process more than a pair of items at a time). Finally, using a more elaborate model, we show that both multiplexing states are computationally useful for the network in solving the so-called “binding problem”, i.e. avoiding the false conjunction of stimulus feature pairs.

### The Model

**Fig 2:**
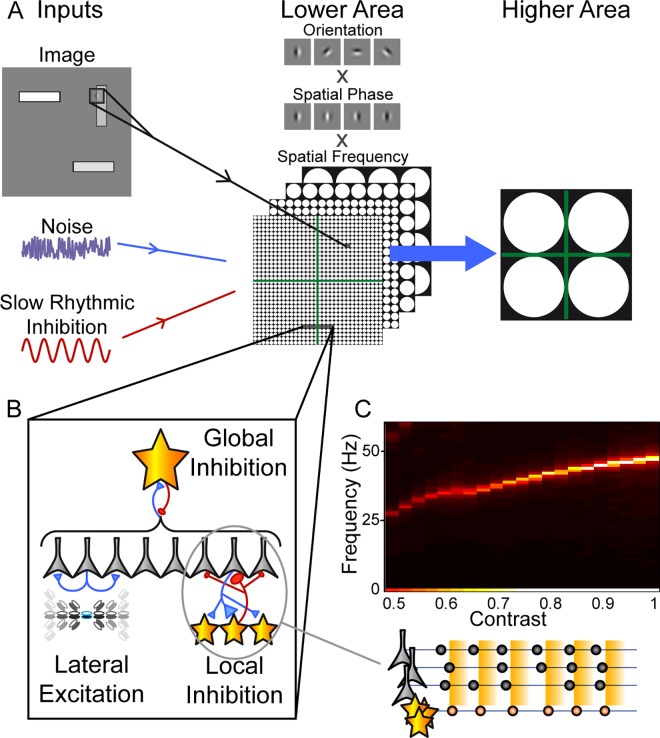
The network model. **A.** The model comprises two areas: a lower area approximating primary visual cortex, composed of multiple retinotopically arranged ‘simple cell’ layers duplicated across orientation, spatial phase and spatial frequency tuning. For input, these cells received a visual stimulus (excitatory conductance driven via an appropriate Gabor filter), noise (independent across cells) and a common slow rhythmic inhibitory conductance to mimic an alpha/theta oscillation (9 Hz in all of our simulations). In turn, the lower area sent convergent connections to a simplistic higher area, comprised in this case of just 4 cells, each receiving input from one retinotopic quadrant of the lower area (i.e. pure spatial selectivity). **B.** Within each area, a number of local circuit mechanisms could be implemented: lateral excitation (see Methods for details); local inhibition with a 2-D Gaussian distribution of synaptic strength both to and from a layer of inhibitory interneurons; and global inhibition implemented via a single interneuron receiving input from and sending output to all the excitatory cells in the lower area. In the simulations reported, local excitation and inhibition were always included, whereas global inhibition was varied as an important parameter determining network behaviour. **C**. The power spectrum of activity generated in the lower area in response to a single stimulus object (light rectangle, 9 x 39 pixels, with a single-pixel dark outline) as a function of its contrast, calculated for the PSTH of all activated cells (10 repeats of 1 second of activity for each contrast level tested; note that no global inhibition was included in these simulations). The network readily generates gamma frequency activity, via a PING mechanism as illustrated diagrammatically below the plot (pyramidal cells and their times of firing shown in gray; inhibitory interneurons in orange; the orange rectangles in the background represent the time course of inhibitory feedback). The frequency of gamma activity is contrast dependent, matching experimental results.

The design of our model (Fig 2) was guided by several simple principles. These are detailed below, but in essence, our aim was to construct a network model incorporating several simple recognised characteristics, to test whether, and under what conditions it could generate activity resembling the major multiplexing theories. In short, we included all the ingredients that would seem *necessary* to implement a multiplexing coding strategy. Even then, however, it could be that these separate ingredients are not *sufficient*, and that the network never actually produces a behaviour resembling either of the major multiplexing schemes proposed in the literature. Or it could be that one of these two schemes always dominates the network behaviour, regardless of the parameter regime. Instead, we found that both F- and LJ- multiplexing behaviours could indeed be produced by this network, and that the shift between these modes required only a single change in parameters.

Both the theories under study have been proposed as general computational mechanisms within the cortex, and so we did not set out to simulate a specific system in detail. Nonetheless, we found it useful to be able to present ‘real’ stimuli rather than abstract levels of activation, to more easily understand, contextualize and visualize the results. Thus, we chose to base our model loosely on the early visual system, with a lower area approximating primary visual cortex (V1), and projections to a very basic second area (Fig 2A). Specifically, the lower area comprised a retinotopic layer of 348160 excitatory units with simple-cell like receptive fields (Fig 2A), with lateral excitatory interactions and local inhibitory feedback via a layer of inhibitory interneurons (Fig 2B). Global inhibitory feedback (modelled as a single interneuron receiving input from and sending feedback to all excitatory cells in the lower layer) was included with variable amplitude and was an important parameter in determining the operating state of the network. The higher area was deliberately designed to be simplistic, allowing a clear investigation of the dynamics in the lower area, and how these might establish interesting temporal interactions with a target region. In the first instance, the higher area comprised just 4 excitatory cells, with purely spatial selectivity, each neuron receiving input from one retinotopic quadrant of the lower area (thus each neuron would respond to any stimulus in the corresponding quadrant; after establishing the basic operation of the system, in a second section, we test a more functionally interesting network with overlapping inputs to the higher area). We presented stimuli comprising discrete objects confined to these spatial quadrants, so that in effect, each neuron in the higher area responded to just one object. Note that the network need not be interpreted as simulating the entire visual field but rather a functional region defined, e.g., by the range of overlap of receptive fields in the higher area: cells with non-overlapping receptive fields can behave independently, and no multiplexing process is required at that scale. The global inhibition that we refer to in both lower and higher areas should be understood in this context, as global to the functional region but not necessarily global to the whole anatomically defined brain area (e.g. V1 or V4).

Temporal dynamics within the model are critical to its behaviour in the current context and so we chose to simulate a spiking neuron network with cellular and synaptic parameters drawn from the experimental literature (see Methods). Particular attention was paid to the membrane and synaptic time constants, and net conductance values during activity.

The stimuli used comprised images with a mid-gray background, and from 1 to 4 discrete objects, each constrained to a different spatial quadrant.

### Oscillatory Activity

The kind of excitatory-inhibitory network that we have implemented will readily generate oscillatory activity via a Pyramidal-Interneuron Gamma (PING) mechanism [28,29] (diagrammatic representation in Fig 2C). Pyramidal cell activity activates the local inhibitory network (orange stars in the diagram). Inhibitory feedback from these cells (orange background) shuts down the pyramidal cell activity. As this inhibition decays, the pyramidal cells begin to fire again in a synchronized manner (in our network, this synchrony is enhanced by local lateral excitation, so that the early-firing cells within an ensemble increase the probability that other members will fire soon afterwards). This burst of firing sets in motion the next cycle of excitation-inhibition. With physiological membrane time constants and synaptic dynamics, this system naturally generates rhythmic activity in the gamma frequency range (Fig 2C). Furthermore, there is a growing understanding of the stimulus dependence of gamma generation in the visual system, such that, e.g., gamma frequency increases as a function of stimulus contrast [30,31]. Again, this behaviour arises automatically in a PING network, because more strongly driven pyramidal cells recover more quickly from inhibitory suppression, illustrated for our network in Fig 2C. We were thus able to set the contrast levels used so as to drive gamma oscillations over a realistic frequency range. Simultaneous presentation of several objects with different contrast levels thus resulted in the generation of localized gamma oscillations at different frequencies for each ensemble (51 Hz for a full contrast object vs. 43 Hz for contrast = 0.8; note that this describes the situation with no global inhibition included).

Regarding the slow oscillation, generation of the hippocampal theta oscillation is complex and at least partially driven by input from external structures [32]. Similarly, the cortical alpha oscillation likely involves thalamocortical interactions rather than a mechanism purely intrinsic to the local cortical network [33]. To preserve simplicity, we thus opted to implement the slow oscillation as an external input, a rhythmically modulated conductance directly applied to all pyramidal cells in the lower area (in the results reported here, no slow oscillation was applied to the higher area). An inhibitory conductance was used, again based on the available physiological data: hippocampal theta activity, at least at the soma, is thought to be inhibitory [34,35]; alpha activity in the cortex tends to increase with inattention and decrease with attention, and is again considered likely to be inhibitory [36]. We set the amplitude of this oscillation such that, at its peak, it completely suppressed firing in the lower layer (comparable to the modulation of single units in hippocampus, e.g. [37]). We used a slow oscillation frequency of 9 Hz in the results shown here, intermediate between the theta and alpha frequency bands of interest.

### Functioning States of the Network

At this point we began to introduce and explore the additional features required to address the cross-frequency multiplexing theories.

We carried out a series of simulations (5 repeats of 2 seconds of simulated activity for each condition), exploring a set of parameter spaces (the amplitudes and decay time constants of synaptic connections between the various neuronal populations in the network).

The stimulus comprised a 128 x 128 pixel image (mid-gray background), with an object in three of the four quadrants, specifically white rectangles (9 x 39 pixels), with a single-pixel wide black outline, with contrast levels 1 (top left), 0.95 (bottom right) and 0.9 (top right). Note this simplifying limitation in the model, representing inputs with an unrealistically limited range of absolute contrast levels. However, the visual system includes well studied mechanisms of adaptation and contrast normalization dependent on stimulus statistics (e.g. [38]) such that the kinds of responses described here would actually be possible over a much greater range of contrast values.

In presenting the results from these simulations, we use a pair of measures (see Methods) that characterise the functioning states of the multiplexing modes under study, and convert these to a colour scale: 1) spike separation, the temporal separation of activity driven by the different stimulus objects (green for poor spike separation, i.e. activity is synchronous between the different objects, an indication of failure to implement either theory), and 2) selectivity for a single object (F-multiplexing, red) vs sequential activation of different objects (LJ-multiplexing, blue).

**Fig 3:**
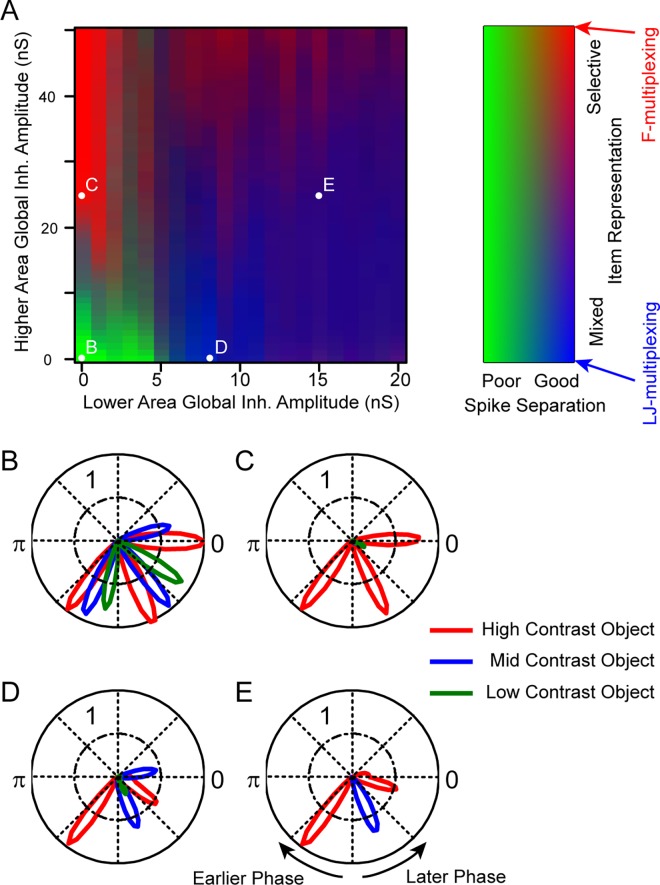
Network states as a function of global inhibition amplitude in the lower and higher area. **A.** The colour map shows the network state dependence on parameter values (other parameters set to default values except as stated, 5 repeats of 2 seconds of simulated activity for each condition), with green reflecting poor spike separation, red selective representation of a single item (F-multiplexing), and blue alternation between different items (LJ-multiplexing); see Methods for details of the indices used to measure spike separation and item representation. **B–E.** Polar plots of higher level activity, spike probability (across simulation repeats) as a function of slow oscillation phase. Colours (red, blue and green) indicate spikes driven by the high, mid and low contrast objects respectively. The different plots correspond to the different network states, as marked in panel A. **B.** Without global inhibition in either area, all items were represented but there was poor spike separation (green area in A). **C.** With global feedback inhibition only in the higher area, the network displayed F-multiplexing, with a single item repeatedly represented at gamma frequency within each slow cycle (red area in A). **D.** The inclusion of global feedback inhibition in the lower area instead yielded LJ-multiplexing, with alternation between different items (blue area in A). **E**. With global inhibition in both areas, the network tended to generate fewer spikes, skipping some of the available gamma cycles. Multiplexing tended to be LJ-style.

Fig 3A presents the resulting network states for a pair of key parameters revealed by these simulations, the amplitude of global inhibition in the lower versus the higher area (all other parameters fixed in this plot). With low levels of global inhibition in both areas (Fig 3A, green area), spike separation was poor, as is apparent in the phase histogram of higher area activity (Fig 3B). In other words, the model did not produce any interesting multiplexing behaviour. With increased global inhibition in the higher area (Fig 3A, upper left red area), the network entered an F-multiplexing state, with selective activation of a single higher level neuron (responding to the highest-contrast stimulus object) at gamma frequency (Fig 3C). Alternatively, with increased global inhibition in the lower area only (Fig 3A, lower right blue area), the network entered an LJ-multiplexing state, with higher area firing alternating between the high- and mid-contrast stimuli (Fig 3D). With global inhibition in both areas, overall firing tended to be reduced as some gamma cycles were skipped, and so encoding in this parameter range may be considered less efficient. Nonetheless, the network state tended towards LJ-multiplexing, with firing alternating between different stimulus objects (Fig 3E).

### Detail of the Different Activity States

**Fig 4:**
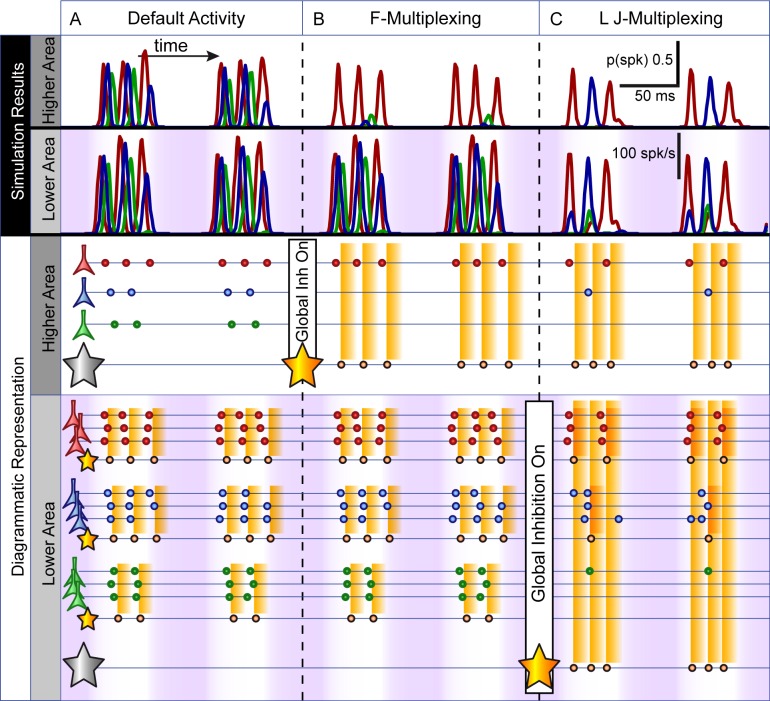
F- and LJ-style multiplexing: simulation results and diagrammatic representation. This figure shows the results of a simulation (100 repeats) in which key parameters were changed over the course of the simulation in order to illustrate and explain the different network states highlighted in Fig 3. Simulation results (top of the Fig) show post-stimulus time histograms (PSTHs) for cells in the upper and lower areas, averaged over 100 repeats of the same simulation parameters. The stimulus image was that shown in Fig 2, comprising three discrete objects of different contrast values (see Methods). Parameters were fixed at default values (Fig 3B), except that at successive fixed time points, global inhibition was turned on, first in the higher area (Fig 3C) and then in the lower area as well (Fig 3E). Red traces are for the cells responding to the highest contrast object (averaged over all the active cells in the relevant quadrant of the lower area; and showing the probability of firing at a given time point for the single cell in the higher area responding to that object), and similarly blue for the mid-contrast object and green for the low-contrast. The purple background in the lower area shows the amplitude of the slow oscillatory inhibitory conductance. The same colours are preserved in the diagrammatic representation of activity. As in Fig 2, inhibitory interneurons, their time of firing and the time course and spatial extent of this inhibitory feedback are indicated in orange. **A**. With no global inhibition in either lower or higher area, activity driven by the three stimulus objects generates independent gamma oscillations in the lower area, and the higher area simply follows this activity. However, the output is not well temporally segregated. **B**. Global inhibition is added to the higher area. On each slow cycle, the first-arriving burst of inputs to the higher area drive a cell which in turn competitively inhibits the other higher area cells. That inhibition decays just in time for the same cell to be driven by its next burst of inputs from the lower level (i.e. a PING gamma is set up in the higher area, matching the dynamics of the gamma in the lower area, and selective communication is achieved, or F-multiplexing). **C**. Global inhibition is added to the lower area. On each slow cycle, when the first ensemble fires competitive inhibition disrupts the activity of the other ensembles. The second ensemble recovers, yielding a second cycle of inhibition. By that point, the first ensemble has recovered and fires again, and the third ensemble never has the chance to fire. Effectively, the independent gamma activities in the lower area are disrupted and replaced by a single global gamma, with activity of just a single ensemble on each cycle.

To help to understand the resulting changes in activity in the lower and higher areas, and how these brought about the different activity states, Fig 4 shows the results of a simulation (100 repeats), along with a diagrammatic representation, in which the changes to global inhibition were made over the course of the simulation run (parameters corresponding to Fig 3B, 3C and 3E). We first set out a point-by-point summary of the key ideas emerging from this simulation, and then describe them in more detail below:

1. In the lower area, with only local inhibition included (i.e. no global inhibition) the stimulus objects each engage an independent gamma oscillation, phase offset from each other dependent on the level of drive (contrast or input gain). On its own, this temporal separation is inadequate to yield good spike separation in the higher area.
2. By including global inhibition between cells in the higher area, feedforward activity engages a gamma oscillation there, the phase of which is automatically correctly set to enable the processing of a single selected object, and to exclude activity relating to the non-selected objects (F-multiplexing).
3. Including global inhibition in the lower area disrupts the independent gamma oscillations, and instead yields a single global gamma oscillation. The global inhibition also results in competition between ensembles, so that only a single stimulus object is represented on each gamma cycle (LJ-multiplexing). The higher area can simply follow this activity.

#### Default mode

Initially (Fig 4A), there was no global inhibition in either area. As the slow oscillatory inhibition decayed, activity in the lower layer arose in a temporally structured fashion, starting with a burst of firing from the ensemble responding to the most strongly driven (highest contrast) object (red PSTH trace; cluster of red cells in the diagram). This was followed in quick succession by similar bursts for the other objects of successively lower contrast (blue and green traces and cells). Each of these ensemble bursts set in motion a localized PING mechanism, yielding independent localized gamma activity at slightly different phases and frequencies for each of the stimulus objects.

Activity in the higher layer of the network simply followed that in the lower area in a feedforward manner, so that the different neurons were each active at gamma frequency, with a slight phase separation in their activity. Note however that there was no overlap between connections to the higher area in this simplistic network. We will show later that the temporal separation here would be insufficient to disambiguate the signals for further processing were convergence included (hence the poor spike separation index for these parameters in Fig 3A).

#### F-multiplexing

Next (Fig 4B), global inhibition was introduced to the higher area, effectively a competitive inhibitory interaction between these cells. This inhibition was rapidly activated by the first cell to respond in the higher area with the result that the neighbouring cells were suppressed and no longer responded to the bursts of excitation arriving from the lower area. However, because inhibitory synaptic time constants were similar across the two areas, inhibition in the higher area decayed sufficiently *just in time* for the next burst of input from the same lower-area ensemble, so that the same cell in the higher area responded again, and again suppressed its neighbours for a short period. Effectively, feedforward activation of the higher area set in motion a locally generated gamma oscillation, the inhibitory phase of which excluded further processing of all but the selected input (the one that produced the earliest firing in the lower-level area). The principle at work here has been described in a model by Börgers and Kopell [39], showing that a target network including feedback inhibition with physiological dynamics will selectively entrain to synchronised input at gamma frequency, excluding distractor inputs. The current results extend this by showing that the required input activity (gamma synchronised, and with temporal offset between the activity of different ensembles) is naturally generated by a simple lower area network. Functionally, this is precisely the same as the CTC mechanism proposed by Fries [16,17], and matches the interpretation of related experimental results [18]. This pattern of activity lasted for a few gamma cycles and was interrupted by the slow oscillatory inhibition, then repeated on the next active phase of the slow cycle.

#### LJ-multiplexing

Finally, (Fig 4C), global inhibition was introduced to the lower area (without, here, removing the global inhibition in the higher area), yielding a switch from independent local gamma oscillations to a single global gamma oscillation. Again, with the decay of the slow oscillatory inhibition, activity began in the lower area as before, with firing in the most strongly driven ensemble. This burst of excitation drove global feedback inhibition, thereby influencing the activity of the other ensembles. Firing in the second ensemble was delayed rather than blocked, so that it occurred on the second cycle of an area-wide gamma oscillation. Again, this drove global feedback inhibition that delayed the activation of the next ensemble–in our network and with the specific stimulus parameters used, the first ensemble was sufficiently recovered at this point that it fired again, interrupting activity in the third (lowest contrast) ensemble. In fact, the third ensemble never had the chance to fire, being repeatedly blocked by the alternating activation of the first and second ensembles (but see below for a simulation with cycling between all 3 items). As above, this pattern of activity was interrupted by the slow oscillatory inhibition, then repeated on the next cycle.

Note that, as Fig 4 highlights, the switch from F- to LJ-multiplexing, selective to exploratory processing, can be reduced to a single parameter change–the inclusion of global inhibition in the lower area.

### F-multiplexing–The Slow Oscillation

**Fig 5:**
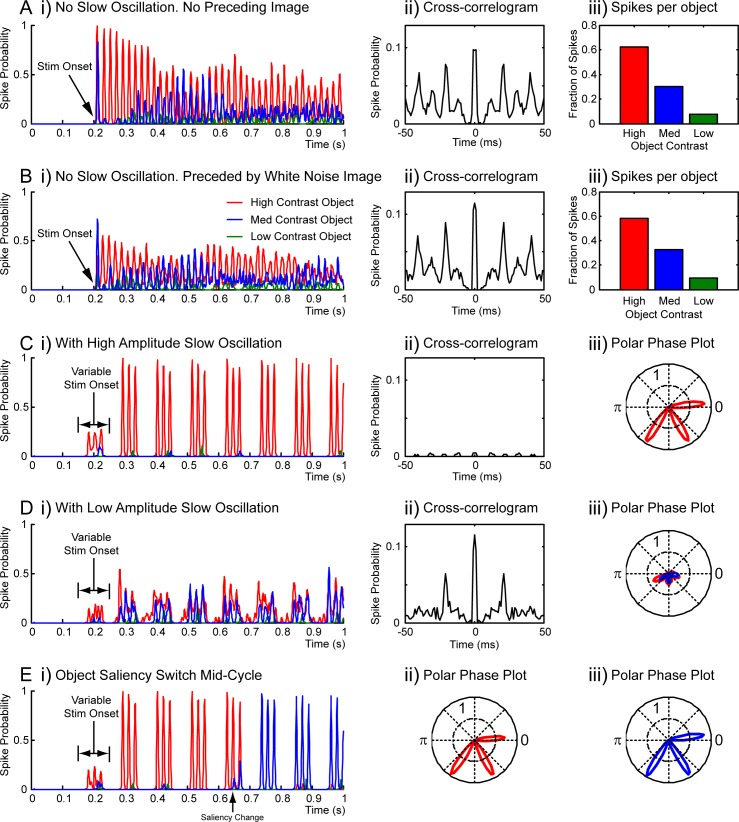
F-multiplexing–the slow oscillation. Results from several series of simulations (100 repeats each), summarizing activity in the higher area (red for high contrast object, blue for mid, green for low). **A.** Without the slow oscillation to rhythmically reset the system, selection via the CTC mechanism fails over time, as the independent gamma oscillations in the lower area shift in and out of synchrony (because of their slightly different frequencies). Parameters as for Fig 3C (i.e. strong global inhibition in the higher area), except that no slow oscillation was included. Following 200 ms of blank screen, the stimulus (3 objects, see text) was turned on. i) shows a PSTH (spike probability across simulation repeats), ii) is a cross-correlogram (calculated for spikes from all 3 cells), showing the poor temporal separation of activity (the large central peak shows that different higher area cells often spike near-synchronously), iii) shows the fraction of spikes driven by each object over the whole simulation period. **B.** Compared to A, selection is even worse when the network starts from a properly randomised state at stimulus onset. As for A, except that the blank screen at the start was replaced by pixelated white noise. **C.** Including the slow oscillation (with amplitude high enough to suppress lower area firing) yields good selection, well maintained over time, and with a predictably structured temporal output even in the face of variable stimulus onset times. As for B, except that the slow oscillation (9 Hz) was included. Also, stimulus onset was random (equal probability) from 150 to 250 ms. iii) shows a polar phase plot of firing probability relative to the slow oscillation. **D.** As for C, except that the amplitude of the slow oscillation was halved, such that some lower area cells were never fully suppressed. As a result, lower area synchrony was disrupted, and object representation/segregation was poor. **E.** The system is also able to switch selected object over time, following a change in stimulus, or, e.g., top down attention. As for C, except that objects of equal contrast (value 1) were used, and input gain varied to mimic top-down spatial attention. Initially, gain was set to match the previously used contrast values (1, 0.95 and 0.9 for the different objects), but after 645 ms, it was switched to prioritise the second object (i.e. 0.95, 1 and 0.9). ii) and iii) show polar phase plots for before and after the gain change respectively.

The slow oscillation has two hypothesised roles in the F-multiplexing system: 1) a regular breaking of gamma synchrony to potentially allow the processing of different items across slow cycles [16,17], and 2) a regular reset of activity, central to the selection mechanism, and important to avoid the different gamma oscillations cycling in and out of phase with each other (because of their slight differences in frequency) [18]. In Fig 5, we show the results of a set of simulations (100 repeats each) that support these conjectures. In the following, the ‘selected’ object always refers to the highest contrast stimulus.

The first simulation shows the higher area activity when the slow oscillation was excluded (Fig 5A). A blank screen was presented for the first 200 ms, at which point the stimulus was turned on. The activity of the 'selected' object was favoured for the first hundred ms, but after that, selectivity was compromised (compare to the robust selectivity obtained when the slow oscillation was included, Fig 5C). The proportions of spikes for each object over the whole time period are shown in the histogram at the right. Looking at the cross-correlogram for the activity, it is apparent that spikes for one object were often closely followed (second spike within 0 to 3 ms for 20.8% of spikes) by spikes for another. That is, spike separation was compromised, exactly as predicted because the lower area gamma oscillations drift in and out of phase over time (indeed, this drift in phase difference is directly apparent in the wave of responses to the medium contrast object from about 300 to 600 ms).

The activity was even further compromised in the second simulation (Fig 5B), where rather than a blank screen, random pixelated white noise was presented for the first 200 ms, so that neurons were in a variable state at stimulus onset. In that case, activity for the 'selected' object was scarcely favoured in the early period, and spike separation was even more compromised (second spike within 0 to 3 ms for 25.3% of spikes).

In the third simulation (Fig 5C), the slow oscillation was restored, and as well as the noisy pre-stimulus period as above, a random delay was introduced to the timing of stimulus onset (equal probability from 150 to 250 ms). This effectively randomized the phase of the slow oscillation at which the stimulus occurred. In spite of this variability, very strong selectivity was established from the next slow oscillation cycle following stimulus onset, and maintained throughout the simulation period.

The fourth simulation (Fig 5D) was similar except that the amplitude of the slow oscillation was halved, with the effect that some cells in the lower area were never fully suppressed. As a result, when slow oscillatory inhibition fell, the near synchronous bursts of activity for each object were disrupted (because the most strongly driven cells were refractory, and not available to participate in the ensembles), and communication with the higher area was impaired. Note the paradoxical effect, that decreased inhibition in the lower area led to decreased activity in the higher area.

In the fifth simulation (Fig 5E), we tested the ability of the system to switch selected object. The initial period was as described for the third simulation (pixelated noise, and variable stimulus onset time), but in this case each of the three stimulus objects was presented at full contrast, and instead the input gain for each quadrant (which we conceive as the implementation of a top-down selection mechanism) was modified: initially, it was set to match the previously used contrast values (1, 0.95 and 0.9 for the top left, bottom right and top right quadrants, respectively), but after a delay (three and a half cycles of the slow oscillation), the values for the first two quadrants were switched (mimicking, e.g. a change in top-down spatial attention). The network readily followed this change in selection, representing the top left object up to the time of the gain change, and then switching to the bottom right object. Note that, even though the saliency switch occurred mid-way through the active part of the slow cycle (arguably the most challenging situation for the model), object selection was rather resistant to that interruption: representation of the previously selected object continued until the end of the cycle, with only a small amount of activity relating to the newly prioritised object leaking through. As predicted theoretically, the switch to representation of the new object occurred on the following slow cycle (following breaking of inter-area communication by the slow oscillation). Beyond this, it is apparent that, in the brain, abrupt stimulus changes result in phase resetting of slow oscillatory activity [e.g. 40,41], likely enabling a rapid switch of attention to the salient event.

### LJ-multiplexing–Representation of Multiple Items

**Fig 6:**
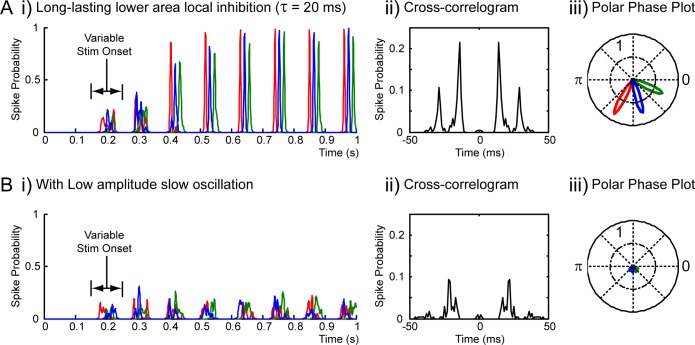
LJ-multiplexing–representation of multiple items. **A.** The system is capable of representing more objects (more closely matching the proposals of the LJ-multiplexing theory), provided that ensembles auto-inhibit for a longer period, and therefore fire only once per slow cycle. Results from a series of simulations (100 repeats), summarizing activity in the higher area (red for high contrast object, blue for mid, green for low). Parameters as for Fig 3D (i.e. strong global inhibition in the lower area), except that the synaptic decay time constant for local inhibition in the lower area was increased to 20 ms. Following 200 ±50 ms of blank screen, the stimulus (3 objects, see text) was turned on. i) shows a PSTH (spike probability across simulation repeats), ii) is a cross-correlogram (calculated for spikes from all 3 cells), iii) shows a polar phase plot of firing probability relative to the slow oscillation. **B**. As for A, except that the amplitude of the slow oscillation was halved, such that some lower area cells were never fully suppressed. Again (c.f. F-multiplexing, Fig 5D), lower area synchrony was disrupted and object representation was impaired.

In the simulations presented above (Figs 3 and 4), it is apparent that for the LJ-multiplexing style, only two out of three objects are represented, whereas in the original theory a sequence of up to seven or more objects, one per gamma cycle, could be repeated or updated on each slow cycle. As Fig 4C makes clear, the main barrier to multiple items being represented in the network is that, following their activation in a given gamma cycle, lower area neurons recover during the following cycle, and are sufficiently ready to activate again in the cycle after. Thus, rather than an ordered sequence of activations (ABC), the network alternates between the two most strongly driven ensembles (ABA…) and the weaker ensembles never get a chance to fire. One obvious solution is for cells to be suppressed for a longer period following their activation, either by intrinsic spike-induced conductances (e.g. Ca-activated K currents, [42]) or by longer-acting local feedback inhibition. We tested this idea (Fig 6A), using a simulation (100 repeats) with identical parameters to Fig 3D, except that the synaptic decay time constant for local inhibition in the lower area was increased from 3 ms to 20 ms. In that case, the network readily cycled through the three presented objects on each cycle of the slow oscillation. Note that the original formulation of the theta-gamma coding theory allowed for the representation of 7 or more items, consistent with ideas about verbal working memory, and the frequency ratio of gamma and theta oscillations in the hippocampus [6], whereas the lower ratio of gamma to alpha frequencies has led to representation of only three or four items being proposed for the visual system [9,11]. The exclusion of low-saliency ensembles from the multiplexing sequence is actually an explicit prediction of the LJ-multiplexing theory [9,11].

Similar to F-multiplexing, we also tested whether LJ-multiplexing was dependent on complete suppression of activity during part of the slow oscillation cycle. Fig 6B shows the results of simulations, similar to Fig 6A, except that the amplitude of the slow oscillation was halved. Again, synchrony in the lower area was disrupted, and communication of activity to the higher area impaired. Two specific points are worth noting: i) the ordered representation of items (highest contrast first), characteristic of LJ-multiplexing is also disrupted, predictably given that the transform from excitatory drive to phase of firing is known to be dependent on complete suppression of firing for a brief period [13]; and ii) from the cross-correlogram of activity for the different items (Fig 6Bii), items are not represented simultaneously, but rather at discrete gamma cycle intervals–that is, the mechanism of item segregation via global inhibition in the lower area is robust to the loss of structuring brought about by the reduction in slow oscillation amplitude.

### Feature Convergence and Signal Separation

One of the principal computational advantages offered by ‘communication-through-coherence’ is the ability to maintain separation of stimulus signals in the face of substantial convergence and generalization in the processing hierarchy [16]. This can be seen as a partial solution to the infamous “binding” problem [43]. The temporal separation of representations achieved by LJ-multiplexing is potentially useful for the same goal. In order to determine whether the multiplexing modes described really do offer this computational utility, we tested a slightly modified version of the model, as follows.

The challenge in this case is to have cells in the higher area that are selective for specific conjunctions of stimulus features but that will not give rise to ‘illusory conjunctions’, that is, be erroneously activated if the requisite features are present in the stimulus display but spread across multiple objects (bearing in mind the growth in receptive field size along the processing hierarchy as part of the increasing generalization). Starting from the orientation selectivity already intrinsic to the lower area, we added a second simple feature channel, that of colour selectivity. Again, this is not intended to reflect the detail of an actual mechanism in visual processing (it is clear that processing of colour and orientation are not truly separated in primary visual cortex, [44]) but rather a conceptually clear implementation of this general class of computational problem (i.e., feature binding).

**Fig 7:**
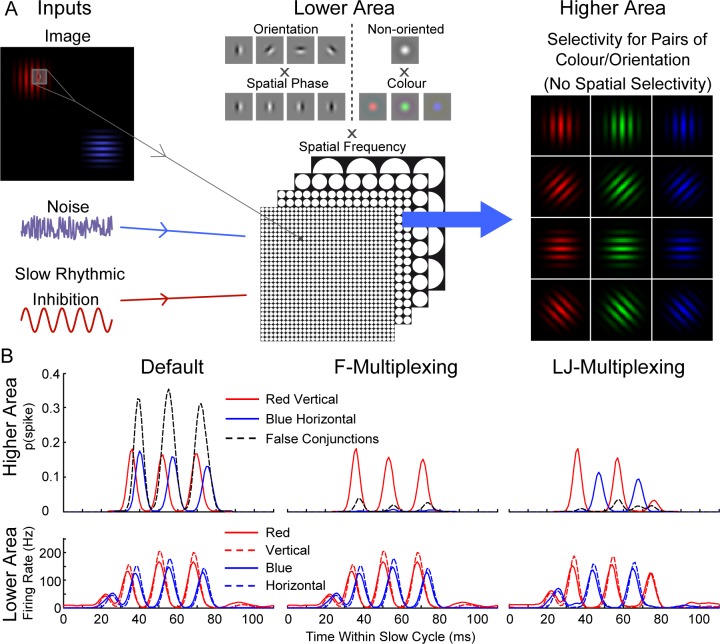
The feature-pair conjunction model. **A.** The lower area of the model is similar to the first model (Fig 2), with retinotopically arranged layers of excitatory and inhibitory cells, but with the addition of layers of excitatory cells selective for stimulus colour (red, green and blue), again duplicated across spatial scales, but without orientation selectivity. The higher area is more significantly modified: in this case, there is no spatial selectivity, with all cells receiving inputs from the full spatial extent of the lower area. Instead, due to restricted connectivity, these cells are selective for particular colour-orientation conjunctions, as indicated diagrammatically. **B.** Summary of network activity in response to a red-vertical stimulus patch, and a slightly lower contrast blue-horizontal patch, across the same three network states as described earlier (default, F- and LJ-multiplexing), showing the activity across one slow oscillatory cycle for each state (average of 1 s simulated activity, 100 simulation repeats). Plots of mean firing rate vs phase of slow oscillation are shown for activity in the lower area. For the higher area, the probability of firing at a given time point is shown, for each of the correctly activated conjunction cells (red-vertical and blue-horizontal) as well as for either of the false conjunctions (red-horizontal or blue-vertical).

In detail, retinotopic layers of colour-selective cells (non-orientation selective) for red, green and blue were added to the lower network area, precisely in parallel with the existing orientation-selective layers, and likewise duplicated across spatial scales (Fig 7A). Connections to and from the local inhibitory network were exactly as for the earlier model, and did not differentiate between the orientation-selective and colour-selective layer (that is, activity in e.g. the orientation-selective cells could lead to local inhibition of the colour-selective cells).

The higher area was more substantially modified. Spatial selectivity was removed, such that all cells received input from the full spatial extent of the lower area. Instead, cells were made selective for specific colour-orientation conjunctions (e.g. vertical-red). This selectivity was established simply through targeted connectivity from the lower to higher area (e.g., the vertical-red cell receiving input only from the vertical- and red-selective cells in the lower area), along with tuning of the synaptic strength such that activity in both input channels was required to drive the cells to threshold. One cell for each orientation-colour pair was included in the higher area (thus, a 4 x 3 grid of cells, see Fig 7A).

We challenged this network with a stimulus comprising two objects with different colour-orientation features (e.g. vertical-red and horizontal-blue). It was straightforward to demonstrate that in a pure feedforward excitatory version of the network (all local and global inhibitory activity turned off) such a stimulus could successfully drive the relevant conjunction-selective cells in the higher area (vertical-red and horizontal-blue) but would also yield illusory conjunctions, by erroneously driving two cells selective for conjunctions not actually present in individual stimulus objects: vertical-blue and horizontal-red.

Fig 7B presents the activity in the lower and higher areas for the three more elaborate network states presented above (c.f. Figs 3 and 4). First, we considered the default activity state (c.f. Fig 4A; slow oscillation included, localized gamma activity generated intrinsically through a PING mechanism in the lower area). Note that we presented the vertical-red object at higher contrast than the horizontal-blue (1 vs 0.95), and so there was some degree of temporal separation between the bursts of synchronized activity in the lower area corresponding to each of the objects (as above, the more strongly driven representation was activated earlier on decay of the slow oscillatory inhibition). However, as is apparent in the upper panel, this slight temporal separation was inadequate to segregate the signals for the purposes of selective representation in the higher area: cells selective for false conjunctions (vertical-blue and horizontal-red) were strongly activated. Following the arrival of the burst of red/vertical inputs, synaptic and membrane time constants were sufficiently long to allow subsequent summation with the burst of blue/horizontal inputs, yielding the false activations.

We then considered the F-multiplexing state (Fig 7B, middle panel), implemented as above via the inclusion of feedback inhibition between the different cells in the higher area. The underlying activity in the lower area was completely unchanged, thus the first major event on decay of the slow oscillatory inhibition was again a synchronized burst of activity in the red-selective and the vertical-selective cells, which duly drove the vertical-red selective cell in the higher area. This activation led to inhibition of the other cells in the higher area, so that they were unresponsive when the second (horizontal-blue) burst of activity arrived: by selectively processing the most salient item only, other representations including the false-conjunctions were successfully excluded.

Finally, we considered the LJ-multiplexing state (Fig 7B, right-hand panel), with a global inhibitory interaction between ensembles in the lower area. In this case, early in the slow oscillatory cycle, we observed the same red-selective/vertical-selective synchronized burst as noted above. However, the resulting inhibitory feedback to the other lower-area ensembles delayed the next synchronous burst (blue-selective/horizontal-selective cells). As a result, although this burst successfully activated the blue-horizontal conjunction cell, it arrived too late in the higher area to be summated with the earlier red-vertical burst, and no false conjunctions were generated.

In summary, both F- and LJ-multiplexing states enabled a temporal labelling of item-specific stimulus information such that erroneous mixing of characteristics was avoided, even in a higher processing area with significant spatial overlap of inputs.

**Fig 8:**
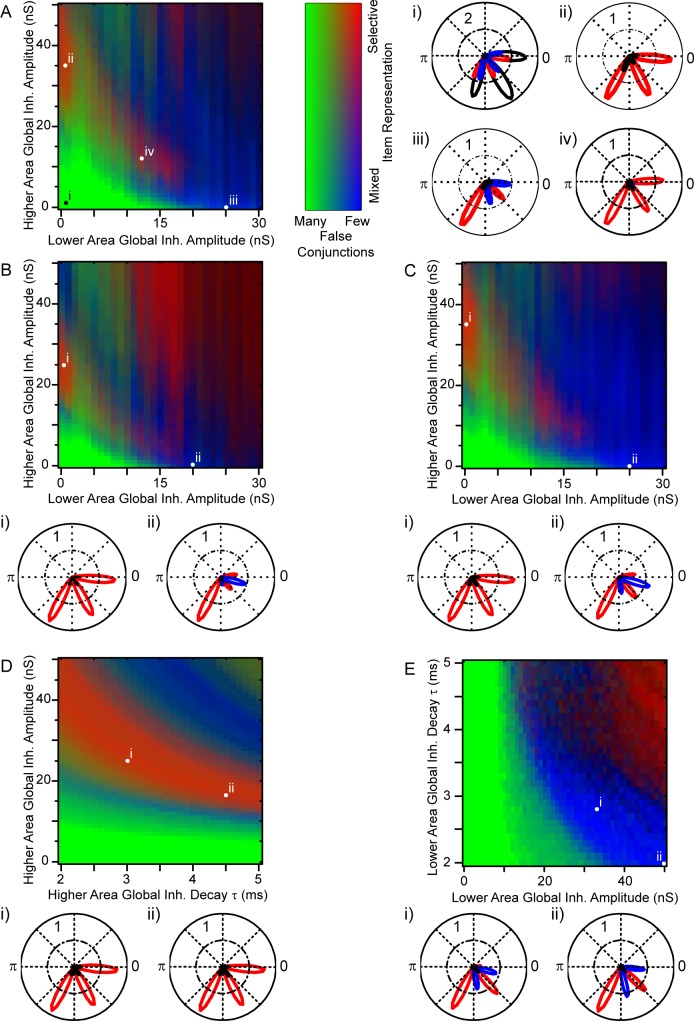
Robustness of network states to parameter changes. Colour maps show the network state as a function of pairs of parameters (other parameters set to default values except as stated, 5 repeats of 2 seconds of simulated activity for each condition), with green reflecting poor performance (many false conjunctions), red selective representation of a single item (F-multiplexing), and blue alternation between different items (LJ-multiplexing); see Methods for details. **A.** With other parameters set to default values, behaviour of the network as the amplitude of global inhibition in the lower and higher areas is varied is similar to that described previously for the first network (Fig 3). Polar phase plots to the right (i–iv) show the actual activity patterns for the indicated parameter values (red, high contrast item; blue, mid-contrast item; black, false conjunctions). **B.** With less feedforward excitation from lower to higher area (120 pS peak synapse amplitude, reduced from 135), the pattern of network states shifts down and to the left. Polar plots below the colour map show activity patterns: the F-multiplexing state is well maintained, but LJ-multiplexing performs poorly, with a tendency to skip gamma cycles. **C.** With the same feedforward excitation as in B, but with the time constants of synaptic decay for global inhibition in the lower and higher areas reduced (to 3.0 and 2.4 ms respectively), something like the original pattern of results in A is restored. **D.** With these parameters fixed, and the amplitude of global inhibition set to 0 nS (i.e. taking a vertical slice through point i in panel C), the F-multiplexing state is seen to be maintained over a broad range of values as the amplitude and decay time constant of global inhibition in the higher area are covaried, that is, the activity is not tightly dependent on precise parameter values. **E.** Similarly, with higher area global inhibition set to zero (taking a horizontal slice through point ii in panel C), the amplitude and decay time constant of global inhibition in the lower area can be covaried and the LJ-multiplexing state maintained.

Fig 8 presents a summary of the parameter space exploration for this network. As above (see Fig 3), the key pair of parameters determining multiplexing style are the levels of global inhibition in the lower and higher areas (Fig 8A). The same results are apparent: that without global inhibition in either area, spike separation is poor (here, leading directly to the generation of false conjunctions); that global inhibition in the higher area yields F-multiplexing; and that global inhibition in the lower area yields LJ-multiplexing. As regards activity with both lower and higher area global inhibition, this network is more seriously compromised, with spikes on many gamma cycles missed. We believe that this arises because the levels of feedforward excitation have to be more carefully tuned in this network version, in order for higher area cells to respond selectively to the presence of both of their input features but not to either feature alone.

The other panels of Fig 8 show other areas of the parameter space, to illustrate the effects of various perturbations.

Fig 8B shows the same parameter pair as Fig 8A, except that the amplitude of the synaptic conductance from lower to higher areas was reduced, from 135 to 120 pA per connection. Essentially, the pattern of results is seen to be similar, but shifted down and to the left, so that lower levels of global inhibition yield comparable results. However, as a result, the area yielding strong LJ-multiplexing was lost. In Fig 8C, the same reduction in feedforward excitation is kept, but the decay time constants for global inhibition have been reduced (from 3.8 to 3.0 ms in the lower area, and from 3.4 to 2.4 ms in the higher area). This has the effect of restoring a pattern of results very similar to the original simulation set (Fig 8A). This is just one example of a large number of manipulations whereby a change in excitation in the network can be offset by a comparable change in inhibition, the point being that the key multiplexing states examined in this study are not dependent on some tightly defined set of parameters, but rather reflect areas of relationships between parameters whose precise values could have been chosen differently.

This lack of dependence on precise parameter values is further emphasised in the lower panels. Fig 8D takes the parameters for the area of F-multiplexing in Fig 8C (i.e. no global inhibition in the lower area), and shows the effect of varying the synaptic decay time constant and amplitude of global inhibition in the higher area. The broad streak of effective F-multiplexing shows that a) again, changing the value of one parameter (e.g. increasing the time course of inhibition) can be offset by changing another (in this case, decreasing its amplitude), and b) even for a fixed amplitude of inhibition, a rather broad range of decay time constants (e.g. ranging from 2 to 4 ms in the centre of the plot) could have been chosen and good F-multiplexing still obtained. Similar results are shown for the LJ-multiplexing state in Fig 8E, where the amplitude and time constant of global inhibition in the lower area are varied (with no global inhibition in the higher area). Thus, perhaps surprisingly for a network dependent on temporal interactions, the time constants of inhibition are not absolutely critical for its operation.

## Discussion

We have used a spiking neuron network model to implement and explore two major theories of cross-frequency multiplexing. In doing so we have confirmed that they are both physiologically plausible and computationally useful. Furthermore, our results have shown that the two multiplexing states are more similar than might intuitively be expected, with a single parameter change, the inclusion of global inhibition in the lower area, able to yield a switch between states. With a second parameter change, in the level of global inhibition in the higher area, more robust state switching is obtained. This leads to the intriguing proposal that both of these multiplexing theories may be correct, reflecting different processing modes for the network.

We should reiterate that, while we have referred throughout to ‘global’ inhibition in both lower and higher areas, the actual spatial scale implied by this must be viewed in context. For example, in our network F-multiplexing requires ‘global’ inhibition between the handful of neurons in the higher area. Obviously, in the real system, this inhibition would only need to extend far enough to include those neurons whose receptive fields overlap with that of the ‘selected’ cell/ensemble, leaving open the possibility that multiple F-multiplexing style networks could be established simultaneously, spatially, across a given network pair. The extent to which this flexibility must be constrained by e.g. top-down attentional mechanisms, or the increasing convergence of connectivity through the hierarchy of sensory processing areas seems a rich question for further study.

### Limitations of the Model

We have constructed our spiking network model guided by physiological parameters. However, the degree of oscillatory synchronization between cells is probably higher here than is generally observed *in vivo* [30,45], and a number of models have specifically addressed the generation of ‘weakly’ synchronised gamma activity [46,47]. We say probably, because estimation of the true degree of synchrony for ensembles is fraught with difficulty, in that large-scale multi-unit recordings in vivo are required, ideally in the awake animal, and even then a simple average level of synchrony is not useful–candidate members of an ensemble must be identified and their activity compared. Beyond even this, one cannot assume that all cells responding to a given stimulus should be considered part of the ensemble–there is no theoretical reason to suppose that all cells within an area should participate in these kinds of oscillation-based processing and already, considerable layer-specificity of both oscillations and synchrony has been demonstrated [25,48,49].

Assuming, however, that our model is too synchronized, we justify this on the following grounds. Firstly, this approach has enabled a clear understanding of how the network works, and what happens as the parameters are modified. Of course, this understanding would be useless if it were founded on false assumptions, and so secondly we would argue that the mechanisms explored here are not strongly dependent on the degree of synchrony. Even with much noisier lower area activity, inhibitory feedback would yield periods of relative synchrony, which could effectively restrict feedforward processing to short temporal windows. The investigation of the network’s robustness to noise is an important potential follow-up of the present work. This will require a more complex model; notably, the higher area of the current network is not well suited to processing noisy inputs, because the very small number of cells included tends towards all-or-nothing responses.

### The Slow Oscillation

In the results presented, we have shown the potential network activity patterns for just a single slow oscillation frequency of 9 Hz, somewhat intermediate between the typically described alpha or theta frequencies. A number of points are worth making with regard to this.

Firstly, the implications of varying the slow oscillation frequency are actually rather straightforward. A decreased frequency in the context of LJ-multiplexing would increase the number of gamma cycles per slow cycle (and there is evidence that the hippocampal theta oscillation frequency can be modulated for precisely this purpose, thereby increasing the number of items processed [50]), but would also increase the delay between system updates. For F-multiplexing, the system is intended to process a single item per slow cycle and so a decrease in frequency would simply allow more iterations of the selected object per slow cycle. Similarly, it would increase the delay between potential system updates.

Secondly, for both of the multiplexing modes presented here, the slow oscillation plays an identical role of regular system reset (in order to allow a system update with a fresh ordered presentation of the salient objects in the LJ case, or to enable selection of a new attended object and prevent the accumulation of gamma-phase errors over time in the F case). It is worth noting that *none* of these mechanisms require that the slow ‘oscillation’ be sinusoidal, or even strictly periodic. Thus the frequency of this oscillation could vary over time according to processing requirements. Equally, any other quasi-regular event, such as visual saccades or microsaccades [51], could perform exactly the same role of system reset, complementary to intrinsically generated oscillations and allowing for a system that can respond more dynamically to behavioural demands.

Thirdly, in both theories, following the slow oscillatory reset, the initial temporal separation for different items arises because of differences in the drive to cells. This leads to the question of what happens when the system is challenged by items generating similar levels of drive? While the theoretical aspects of this question could be usefully modelled, experimental work will be required to establish the behaviour of real biological systems in this regard.

### F-multiplexing–A Selective/Attentional Mode

A number of theoretical studies have considered mechanisms related to the CTC theory [39,52–55]. In particular, Börgers and Kopell [39] showed that the competitive advantage of rhythmically synchronized inputs over less synchronized is greatly enhanced if the target region includes feedback inhibition via GABA_A_-receptor-mediated synapses (effectively establishing an entrained gamma oscillation in the target region), especially if the synchronized input is rhythmically active at gamma frequency. Further, they showed that, even when two input trains have equal levels of synchrony, only one will be selected because of the entrainment established with the target region. This scenario (as distinct from alternative versions relying on different levels of synchrony in selected and distractor inputs) was supported by recent *in vivo* electrophysiological results [18]: two visual stimuli each drove gamma oscillations in V1, and attentional selection of one stimulus did not change the oscillation amplitude but rather induced a slight increase in oscillation frequency. The authors hypothesised that this difference in gamma frequency would allow the selected input to arrive first in the target region (V4) on each gamma cycle, establishing the required entrainment (although, with the difference in gamma frequencies, clearly this situation cannot persist over many cycles, hence part of the need for a slow oscillation to reset activity). This is precisely the scenario apparent in our model. Additionally, Akam and Kullmann [55] have established that for efficient CTC, the selected input must be differentiated from distractors by the amplitude, phase or frequency of its oscillatory modulation: our model shows that the required phase differentiation can be established at the onset of each slow cycle of activity.

As a final observation regarding F-multiplexing, the very act of designing a model to implement this theory led to a simple insight that is nonetheless frequently overlooked in discussions of this system. We have shown that LJ-multiplexing relies on competitive interaction between ensembles, a global feedback inhibition. The flip-side of this observation is that, in systems such as the hippocampus where the individual neuronal elements of a given ensemble are spatially distributed, and thus different ensembles necessarily overlap physically, any inhibitory feedback activity is very likely to be of this global nature–it seems unlikely that inhibitory feedback could be targeted to only the members of a given ensemble in order to generate something equivalent to the local inhibition in our model. On the other hand, we have shown that F-multiplexing is reliant on the *absence* of global inhibitory feedback, that it requires each ensemble to have its own, independent gamma oscillation (Figs 3 and 8). Thus, while F-multiplexing, or communication-through-coherence more generally, can work effectively in spatially structured networks such as the retinotopic visual system, it would be unwise to assume that similar mechanisms could be at play in other, differently structured networks. In short, not all gammas are equal, and we should take great care in generalizing our thinking about these computational mechanisms.

### LJ-multiplexing–An Exploratory Mode

Our model has likewise confirmed the feasibility and computational utility of LJ-multiplexing. The potential of global feedback inhibition between ensembles to yield temporal segmentation of object representations on a gamma cycle-by-cycle basis has already been demonstrated [56]. The new insights provided by our model relate to the inclusion of the slow oscillation.

We have shown that, as predicted, the slow oscillation provides control of timing on a longer time scale, a global temporal scaffold on which to hang the individual gamma cycles in a meaningful way. Thus, the order of activation of the different ensembles directly reflects their ‘salience’, inasmuch as the level of excitatory drive can be considered the interaction of bottom-up stimulus characteristics and top-down modulation of the resulting activity. This transformation of excitatory drive to slow-oscillation phase relies on a well-characterized simple biophysical mechanism [13], and is well supported by *in vivo* experimental evidence [12,35,57].

Beyond this, our model highlights potential limits to the number of objects that can be processed within a single slow cycle. With competitive interaction between ensembles, rather than cycling through all possible stimulus representations [6], the natural tendency of the network is to show repeated activations of the most strongly driven one or two ensembles, with the others excluded. We have shown that multiple items can be represented if, following activation, ensembles are inhibited for a more prolonged period (Fig 6). If we again consider slow oscillations with different frequencies, obviously for a lower end theta oscillation (at e.g. 5 Hz) even longer inhibition of ensembles would be required, although this in itself is not an obstacle to the plausibility of the theory: GABA_B_ receptor dynamics are reported with time constants up to hundreds of ms [*e*.*g*. 58]. The activity patterns recorded in the rodent hippocampus are consistent with precisely this kind of mechanism, with experimental records of the well-known theta phase-precession phenomenon showing not just firing earlier in the cycle, but a reduction in firing late in the cycle [59,60]. However, it is less clear that such mechanisms could be in place in sensory areas like the early visual system–firing rate has a long-established role to play in the sustained coding of multiple stimulus features (contrast, orientation-tuning, etc.) and intuitively, this coding role might be compromised if firing had to be restricted to just a single small gamma-window of opportunity on each slow (~100 ms) cycle (but see [11] for a counter-argument).

### Mode Switching

Our model highlights a striking and unexpected similarity in the network architecture and states that can support the F- and LJ-multiplexing modes. Just a single parameter change, the introduction of global inhibitory feedback in the lower area, was required to switch from F- to LJ-multiplexing, leading us to propose that just such a switch might occur physiologically, underlying a behavioural shift from attentive (F-) to exploratory (LJ-) processing.

In this case, the lower area global inhibitory feedback required for the LJ-multiplexing mode would be associated with increased gamma synchrony over that network population, and thus experimentally, gamma activity measured at the lower network level would actually decrease with attention. Similarly, the gamma phase difference between different lower area recording sites should increase with attention. In the higher area (Figs 3 and 8), the global inhibition/competitive interaction between ensembles, essential for F-multiplexing, is disadvantageous for LJ-multiplexing, and so the switch from exploratory to selective might be accompanied by an increase in gamma in the higher area. This kind of difference between areas in the attention-induced change in gamma amplitude has already been reported: in a comparison of human lateral occipital cortex and fusiform gyrus [61]; and more particularly in local field potentials in the awake behaving macaque, where attention was associated with a decrease in gamma in V1, along with an increase in gamma in V4 [62; but see 48]. While this latter finding seems to support our model, further work will be required to test whether the LFP recordings in question correspond more to the local or the global gamma.

One can imagine a variety of different mechanisms whereby neuromodulatory input could yield an increase in global gamma and thus a network shift from F to LJ-multiplexing, e.g. via the recruitment of a specific interneuron pool with global characteristics (long-range synaptic connectivity, or strong gap-junctional connectivity within the pool), or by modulating the integrative characteristics of the more general interneuron pool. For example, there is considerable interest in the role of cholinergic agonists, both in the context of attention [63,64], and oscillatory synchronization [65]. In the hippocampal slice, cholinergic activation drives a global-style gamma oscillation via excitation of inhibitory interneurons [66], and similar mechanisms may be at play in primary visual cortex, where muscarinic receptors are primarily expressed on interneurons [67,68]. Consistent with our prediction above of different attention-related gamma changes across areas, note that there is a significantly different distribution of muscarinic receptors in V2 [67].

In conclusion, beyond the well-studied role of gamma oscillatory activity in sensory processing [16], there is now ample behavioural evidence in humans of a processing role for both theta and alpha oscillatory neural activity, linked to attentional processes [69,70,41,71]. Furthermore, in addition to the well-known theta-gamma coupling in hippocampal activity [72], recent studies in macaque visual cortex have demonstrated cross-frequency coupling between gamma activity and both theta [18] and alpha activity [25]. These findings emphasize the relevance of the theories of cross-frequency coupling that have developed over the past decade or so [7,16,9,10]. Our model demonstrates the computational utility of these theories when implemented in a physiologically constrained spiking network. Further, by implementing these theories within a single network, we show that they could co-exist in the brain, with a simple, physiologically plausible parameter shift enabling dynamic switching between processing states as required.

## Models

The network model comprised a ‘lower’ and a ‘higher’ region of spiking units, connected in a feedforward manner, processing ‘visual’ input (see Fig 2). Cellular and network parameters were derived from the experimental literature, notably the values determining membrane time constants, that is the capacitance, and the leak and net stimulus-driven conductance values [73–76], and synaptic dynamics [77–80]. In particular, note that the decay time-constant for inhibitory synapses is faster than has typically been used in this kind of model of gamma oscillations [46,39], but that this decision was based on recent recordings showing much faster GABA_A_ decay time constants than had been reported previously (1.9 ms or less), when recordings were made at physiological temperature [80].

### Cells

All cells were implemented as leaky integrate-and-fire neurons, with subthreshold integration of inputs according to the equation,

$$C\frac{dV_{m}}{dt}=g_{leak}(E_{leak}-V_{m})+g_{exc}(E_{exc}-V_{m})+g_{inh}(E_{inh}-V_{m})$$

with capacitance *C*, membrane potential *V*_*m*_, and three distinct conductances *g* with associated reversal potential *E*. When *V*_*m*_ crossed a threshold value, *V*_*th*_, a spike was registered, and *V*_*m*_ reset to a subthreshold value, *V*_*reset*_. The leak conductance, *g*_*leak*_, reflects the resting conductance state and was constant, and *E*_*leak*_ is therefore the resting membrane potential (-66 mV in excitatory cells, -70 mV in inhibitory). The capacitance and leak conductance together determine the resting membrane time constant, τ_*m*_ = *C*/*g*_*leak*_. The excitatory conductance, *g*_*exc*_, was the sum of the relevant time-varying synaptic activities generated within the network, along with, in the lower area excitatory cells only, noise (normal distribution, 1 nS standard deviation) and constant stimulus-driven input (derived from the convolution of the receptive field (2D Gabor filter) of each cell with the relevant part of the stimulus, and scaled by a gain factor, to a maximum of 7.5 nS for an optimally-driven receptive-field at full contrast). This gain factor was constant, except for a single simulation series (Fig 5D), in which the gain was variable across quadrants, mimicking a top-down spatial attention mechanism. Beyond these lower area excitatory cells, other cell classes received a tonic depolarizing conductance, bringing their membrane potential into a working range somewhat closer to threshold (local and global inhibitory cells, 7 nS; higher area excitatory cells, 1.75 nS). The inhibitory conductance, *g*_*inh*_, was the sum of the relevant synaptic inputs and, again in lower area excitatory cells only, the slow-oscillatory inhibition (modelled as a large number of inhibitory synaptic inputs with probability of activation varying as a slow oscillation at the relevant frequency; always 9 Hz in the simulations shown). Individual synaptic inputs were implemented as a step increase in the relevant conductance, decaying exponentially over time with time constant τ_syn_. The parameter values used in the simulations presented are shown in Table 1.

**Table 1:** Cellular parameters for excitatory and inhibitory (including global) cells.

Cells	C (pF)	τ_m_ (ms)	g_leak_ (nS)	V_rest_ (mV)	V_th_ (mV)	V_reset_ (mV)
Excitatory	280	16	17.5	-66	-55	-60
Inhibitory	50	4	12.5	-70	-40	-70

### Network Architecture

Excitatory cells in the lower area had simple-cell like receptive fields, arranged in retinotopic layers (128 x 128 cells in the smallest spatial-scale layers), duplicated across 4 orientation selectivities (vertical, horizontal and two diagonals), 4 spatial phases (see Fig 2) and 4 spatial scales (doubling receptive field size and halving spatial frequency at each scale, so that the highest-scale layers were 16 x 16 cells)–that is, there is one cell per receptive field position/orientation/phase/scale combination. The resulting receptive fields were convolved with an input image to derive the separate stimulus-driven excitatory conductances for each cell. Our intention was not to create a detailed model of primary visual cortex (V1), but rather to have a large block of cells (just under 350,000) with a meaningful spatial arrangement, and each receiving a different but non-arbitrary level of excitatory drive.

‘Local’ inhibitory cells in the lower area comprised a single 128 x 128 layer, corresponding spatially to the retinotopic excitatory cell layers. In addition, a single ‘global’ inhibitory cell was simulated.

In the more abstract higher layer, each neuron really represents the activity of an ensemble, and so we did not include local excitatory interactions between them, and inhibition was similar to the global inhibition in the lower area.

Parameter values for synaptic connections are indicated in Table 2. The reversal potential of all excitatory synapses was 0 mV; and of inhibitory synapses, -75 mV. Recurrent excitatory-excitatory synaptic connection strengths within the lower area were calculated as a function of proximity (2D Gaussian, standard deviation 4 cells; an alternative approach using constant synaptic strength but a Gaussian connection probability with distance may be more physiologically accurate [81,82] but is expected to be mathematically very similar in the current context, and would have the disadvantage of being much more memory intensive computationally) and co-linearity of orientation selectivity [56,83], tending to reinforce responses to contours in the stimulus, specifically as:

$$w_{ij}=e^{-\frac{{\left(\phi_{i}-\phi_{l}\right)}^{2}}{2d\sigma_{i}^{2}}}\times e^{-\frac{{\left(\phi_{j}-\phi_{l}\right)}^{2}}{2\sigma_{j}^{2}}}\times e^{-\frac{d^{2}}{2\sigma_{d}^{2}}}$$

where *w*_*ij*_ is the strength of synapse from neuron *i* to neuron *j*, *ɸ_i_* and *ɸ_j_* are the orientation preferences of neurons *i* and *j* respectively, and *ɸ_l_* is the orientation of a line connecting the centre of the two receptive fields, and *d* its length; the parameters, *σ*_*i*_ and *σ*_*j*_ thus set the tolerance for *ɸ_l_* around the orientation preference of *i* (which decays as a function of distance) and *j*, and *σ*_*d*_ sets the decay with distance. In all simulations reported here, *σ*_*i*_ and *σ*_*j*_ were set to 2 degrees, and *σ*_*d*_ to 4 pixels. Again, the intention is not to implement a detailed model of V1, but to include non-arbitrary recurrent synaptic connections between excitatory cells, with potential implications for the generation of synchronized activity. Synaptic connections to and from the local inhibitory layer had a 2D Gaussian spread (standard deviation 4 cells), and there was no inhibitory-inhibitory connectivity. Note that, although most network parameters were physiologically valid, certain synapses were set to low amplitude for cases where many cells drove just a single cell (excitatory-to-global synapses in the lower area, or the feedforward connections from the lower to higher area), and inversely, other synapses were high amplitude where the number of driving cells was small (excitatory-to-global synapses in the higher area, or global-to-excitatory synapses in both areas).

**Table 2:** Synaptic parameters values. These are indexed according to the connected cells (excitatory E, inhibitory I and global G), e.g. E-I indicates the synapses from excitatory to inhibitory cells. Where relevant, the amplitude indicates the maximum synaptic amplitude between nearest neighbours, e.g. before applying the 2-D Gaussian weighting for distance.

	Lvl 1:	E-E	E-I	I-E	E-G	G-E	Lvl1E-Lvl2E	Lvl 2:	E-G	G-E
Amp (nS)		1.2	0.68	0.62	0.1	0–30	0.11		6	0–30
τ_syn_ (ms)		2	2	3	2	4	1		2	3

### Simulations

Simulations and analyses were carried out using Matlab (*Mathworks*), run on a PC computer (Intel Xeon E5520 processor). Differential equations were solved numerically using the Euler method, with a 1 ms time step. Synaptic conductances were calculated analytically (because they follow exponential decays, it is sufficient to multiply the net conductance for a given synapse type by a constant fraction at each time step). Confirmatory simulations of key parameter states (e.g. Fig 3B–3D) were run at a higher resolution (0.1 ms).

In practice, two simulation modes were used. The first was an interactive mode, via a custom-programmed graphical user interface to control parameters and visualize activity online, whereby it was possible to explore network activity states and directly test the effects of parameter changes. We were thus able to quickly establish approximate parameter sets yielding the kinds of activity patterns described by the two cross-frequency multiplexing theories. This approach was complemented by a batch processing mode (typically, 10–100 repeats of two seconds of activity within the network), allowing a more systematic exploration of the multi-dimensional parameter spaces around the identified parameter sets (up to six parameters were simultaneously explored, while retaining a manageable processing time and visualization of results).

### Analysis

To present activity within the whole network, we calculated peri-stimulus time histograms (Figs 4, 5 and 6) or phase histograms relative to the slow oscillatory activity (Fig 7), showing the average firing rate of all participating cells in each lower area quadrant, or for the higher area, the probability of each cell firing, averaged across simulation repeats. These curves were smoothed for presentation by convolving with a Gaussian, with 2 ms standard deviation.

In other plots, where the activity of the cells in the higher area of the network provides an accessible measure of network performance, we present polar plots of firing probability (averaged across simulation repeats) relative to slow oscillation phase. Again, these plots are smoothed with a Gaussian, with 2 ms standard deviation.

For plots of the network behaviour within a parameter space, we derive a pair of measures that capture the functional state of the network, calculated only with respect to the higher area, the output stage of the network:

1. Temporal Segregation: The key feature defining the successful operation of both multiplexing theories is the segregation of activity for the different stimulus items in the temporal domain. For the second network (Fig 7), by design, the failure of temporal segregation, *S*, can be directly measured as the number of false conjunctions (spike count in higher area cells representing feature pairs not actually present in the stimulus, averaged over simulation repeats, normalised to 3 spikes per slow oscillation cycle, clipped at a maximum value of 1). For the initial network (Fig 2), we assume that spikes in the higher area occurring close together in time would actually reflect a failure of temporal segregation if cells in that area received overlapping inputs. Therefore, *S* is calculated by taking the higher area activity for a simulation run, smoothing in the temporal domain with a Gaussian (3 ms standard deviation), and calculating the shared area across the resulting time series from different cells (averaged across simulation repeats). This value was normalised relative to the value for a known poorly segregated state (no global inhibition in lower or higher areas, Fig 3B). Although *S* is sensitive to the degree of temporal smoothing chosen, comparison with results from the second network supports the validity of this scale. To be clear, *S* ranges from 0 (good temporal segregation, no false conjunctions), to 1 (poor temporal segregation, many false conjunctions).
2. Item Representation, Selective vs Mixed: The two multiplexing theories are differentiated by selective activation of a single cell (the highest contrast/gain stimulus object; F-multiplexing) vs alternation across different cells (at least two and potentially more; LJ-multiplexing). Thus we calculate two values, the mean spike count across simulations of the cell coding for the ‘selected’ item, *A* (normalised to 3 spikes per slow cycle), and of cells coding for the other items, *B* (normalised to 2 spikes per slow cycle).

For presentation, we then convert these 3 values (A, B, S) to a colour scale, by directly calculating red, green and blue (RGB) values. To try to maintain clarity, we allow the temporal segregation measure to dominate: either temporal segregation failed and no multiplexing can occur (strong green) or it succeeded and we can then consider the style of multiplexing (F-multiplexing as red through to LJ-multiplexing as blue). Thus, *S*, *A* and *B* are converted to RGB values (on a 0 to1 scale) as follows:

Green=S

(if spike separation is poor, no multiplexing can occur)

Blue=(1−S)×B

(if other cells than the selected one fire, we might be in LJ-mode)

Red=(1−S)×(A−B)

(if only the selected cell fires, we are in F-mode)

Note that, in practice, *B* was never greater than *A* and so a negative value for Red was never obtained. Also, the scale tends towards black for low spiking rates.
